# Specific elements of the glyoxylate pathway play a significant role in the functional transition of the soybean cotyledon during seedling development

**DOI:** 10.1186/1471-2164-8-468

**Published:** 2007-12-19

**Authors:** Delkin O Gonzalez, Lila O Vodkin

**Affiliations:** 1 Department of Crop Sciences, University of Illinois, Urbana, IL 61801, USA

## Abstract

**Background:**

The soybean (*Glycine max*) cotyledon is a specialized tissue whose main function is to serve as a nutrient reserve that supplies the needs of the young plant throughout seedling development. During this process the cotyledons experience a functional transition to a mainly photosynthetic tissue. To identify at the genetic level the specific active elements that participate in the natural transition of the cotyledon from storage to photosynthetic activity, we studied the transcript abundance profile at different time points using a new soybean oligonucleotide chip containing 19,200 probes (70-mer long).

**Results:**

After normalization and statistical analysis we determined that 3,594 genes presented a statistically significant altered expression in relation to the imbibed seed in at least one of the time points defined for the study. Detailed analysis of this data identified individual, specific elements of the glyoxylate pathway that play a fundamental role during the functional transition of the cotyledon from nutrient storage to photosynthesis. The dynamics between glyoxysomes and peroxisomes is evident during these series of events. We also identified several other genes whose products could participate co-ordinately throughout the functional transition and the associated mechanisms of control and regulation and we described multiple unknown genetic elements that by association have the potential to make a major contribution to this biological process.

**Conclusion:**

We demonstrate that the global transcript profile of the soybean cotyledon during seedling development is extremely active, highly regulated and dynamic. We defined the expression profiles of individual gene family members, enzymatic isoforms and protein subunits and classified them accordingly to their involvement in different functional activities relevant to seedling development and the cotyledonary functional transition in soybean, especially the ones associated with the glyoxylate cycle. Our data suggests that in the soybean cotyledon a very complex and synchronized system of control and regulation of several metabolic pathways is essential to carry out the necessary functions during this developmental process.

## Background

Seedling development in higher plants initiates with the process of germination which takes place by first absorbing water from the soil under appropriate environmental conditions, in a process called imbibition and ends with the protrusion of the radicle and elongation of the embryonic axis [1-3]. Cell metabolism resumes rapidly by initially mobilizing lipids, transcripts and protein compounds synthesized during seed development and stored in the dry seed. Eventually cell division starts, DNA and protein synthesis take place and new enzymes and cellular components are made [3,4]. During soybean [*Glycine max *(L.) Merr] seedling development the radicle emerges from the swollen seed elongating towards the soil where the primary root is developed. Then the hypocotyl develops growing towards the surface pulling out the cotyledons [5]. Emergence occurs when the cotyledons reach the surface over the soil.

Seed germination is a complex adaptive trait of higher plants controlled by a large number of genes and environmental factors [1]. Many molecular and physiological studies have been carried out to identify genes and compounds with important roles during seed germination in *Arabidopsis *[3,6-8], *Brassica *[9], *Medicago *[10] and many other plant species. However, the genetic mechanisms restricted to this important physiological process, as well as their regulation and control, require elucidation [2,1].

The cotyledons contain the nutrients and food reserves that supply the needs of the seedlings during germination and emergence until autotrophic growth commences [5]. Shortly after emergence the hooked shaped hypocotyl straightens out and the cotyledons undergo a physiological transition from mainly a nutrient and food reserve tissue (yellow) to an active photosynthetic (green) tissue. Within the cotyledonary cell during this transition, lipids are initially metabolized in the lipid bodies and fatty acids are translocated to glyoxysomes [11-14]. These specialized peroxisomes assist in breaking down fatty acids by β-oxidation which are then converted to succinate in a series of enzymatic reactions known as the glyoxylate cycle [13-15]. Succinate is then processed in the mitochondria throughout the tricarboxylic acid (TCA) cycle and eventually converted to carbohydrate. Once the lipids are metabolized and the cotyledons undergo greening, the number of glyoxysomes decreases and leaf peroxisomes become abundant to participate with chloroplasts and mitochondria in the process of photorespiration. The cotyledonary physiological transition is a complex process that must be under strict gene control and regulation. To be able to describe and understand the genetic mechanisms involved in the functional transition as well as their regulation and control systems it is essential to first define the global gene expression pattern and its modulation throughout development during this process.

Global gene expression studies such as transcriptome, proteome and metabolome analysis have proven to be excellent tools to dissect plant physiological mechanisms and discover new elements of relevant biological importance [8,10,11,16-19]. Gene expression resources in soybean have been described [20] and utilized to deepen our understanding and knowledge of relevant biological processes including somatic embryogenesis [21], response to pathogen challenge [22], elevated carbon atmospheric conditions [23], and gene identification in mutant lines [24]. Recently, a new set of 70-mer oligos representing the soybean EST collection was defined and synthesized (Illumina/Invitrogen, Inc., San Diego, CA). These oligos were designed to represent the most possible 3' end portion of the corresponding cDNA to be able to distinguish gene family members due to the higher sequence variability within this region.

**Figure 1:**
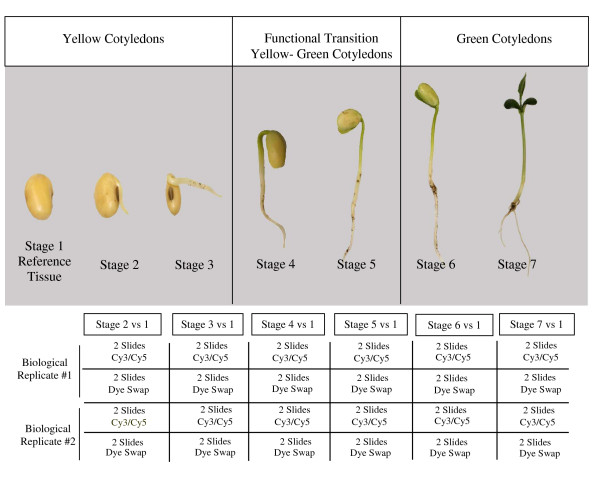
**Experimental design**. Seven sequential developmental stages during seedling development were defined during the time of the study: Stage 1: Imbibed, underground seed for 48 hrs, pre-emerging hypocotyls. Stage 2: Yellow cotyledons, underground, radicle is between 10–15 mm long. Stage 3: Yellow cotyledons, underground, radicle is between 16–25 mm long. Stage 4: Cotyledons start emerging from the ground. They are mostly yellow with green edges. Hypocotyls are 40–50 mm long, green where close to the hypocotyls and yellow where deep in the ground. Stage 5: Cotyledons are yellow and green, above the ground and hanging on the curvature of the hypocotyl. Primary roots are developed. Stage 6: Cotyledons are above ground, mostly green, growing straight from the hypotocyl. Primary roots are developed. Stage 7: Cotyledons are above ground and fully green. Plants are 7–8 cms long with full roots and unifoliolates developed. No trifoliolates. Two technical replicates of 70-mer Oligo chips containing 19,200 features were hybridized to observe differential transcript abundance profile between each of stages 2 through 7 in relation to the imbibed seed (stage 1). The dyes were swapped in two experimental replicates to avoid potential dye bias for a total of 4 Oligo chips per biological replicate. Since two biological replicates were set up, a total of 8 chips were used to compare transcript abundance per germination stage defined.

In this study, we defined the global transcript abundance profile of the functional transition of the cotyledonary tissue throughout seedling development, providing precise identification of the genetic elements that participate in this physiological process, particularly the ones related with the glyoxylate cycle. For this purpose, we defined 7 different developmental stages (time points) starting with the imbibed seed, covering germination and continue with radicle protrusion, hypocotyl elongation, emergence, and development of the unifoliolate (see Figure 1). We identified some of the specific participants of the glyoxylate cycle in soybean cotyledons that are involved throughout seedling development and confirm the fundamental role of these metabolic pathways during the functional transition from nutrient and food reserve to photosynthetic activity. Several other genes with known functions were also identified as key participants as well as unknown genetic elements that have the potential to contribute significantly during soybean seedling development and the cotyledonary functional transition.

## Results

**Figure 2:**
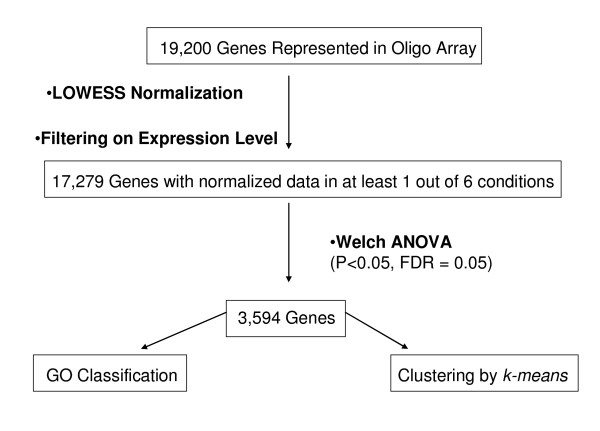
**Flow chart of oligo microarray data analysis**. Microarray data analysis was performed using GeneSpring 7.2, Agilent Technologies (Palo Alto, CA). Data from a total of 48 slides, containing the intensity values for the control and the signal channels as well as the flag values for present, absent or marginal spots was uploaded and sample attributes were defined. After background subtraction and eliminating all the spots flagged as marginal or absent, data was normalized using an intensity-dependent LOWESS (locally weighted scatter plot smoothing) normalization method to minimize systematic non-biological differences and standardize chips for cross-array comparisons. Normalized data was then filtered on expression level to obtain a group of genes with expression values in at least one of the comparisons made. This group of genes was then used for statistical analysis. A Welch ANOVA (parametric test, variances not assumed equal) was carried out with a p-value cut off 0.05, a false discovery rate (FDR) of 0.05 and between 0.1 and 100 fold expression value change. All members of this collection which contain Gene Ontologies classification for plants information were classified accordingly while the ones without it were manually classified using the GO guidelines. Clustering analysis by *k-means *grouped genes with similar transcript abundance profiles during germination and emergence in a predefined number of clusters using standard correlation as similarity measure.

To identify differential transcript abundance during the functional transition of the cotyledon during seedling development, data obtained from scans of 8 oligo chips obtained for each of 6 time-point comparisons (see Figure 1, Methods) was quantified, uploaded into GeneSpring 7.2 (Silicon Genetics, CA), and then transformed, filtered and normalized (see Methods). Time was defined as the only parameter to be taken into account for analysis and 6 different conditions were defined: one for each time point comparison relative to the imbibed seed (see Figure 1). Normalized data was further filtered by expression level for quality control to eliminate genes in the array with no values throughout the time study. Out of 19,200 genes represented in the soybean oligo array 17,279 genes (90%) produced a signal in at least 1 out of the 6 conditions defined (see Figure 2). A Welch analysis of variance (ANOVA) of these genes in 48 samples (corresponding to individual Oligo microarray chips) with a p-value cut off 0.05, a false discovery rate (FDR) of 0.05 and between 0.1 and 100 fold expression change in at least 1 out of 6 conditions, defined a gene collection of 3,594 genes with statistically significant data (see Additional file 1: Complete collection of 3,594 genes with statistically significant data) that was further used for cluster analysis by *k-means *and functional classification of individual members using gene ontologies (see Figure 2). All the results obtained have been deposited in the NCBI Gene Expression Omnibus (GEO) database as accession number GSE6534 and the 70-mer oligo array platform containing 19,200 features as accession number GPL4635.

### Gene classification by gene ontologies

**Figure 3:**
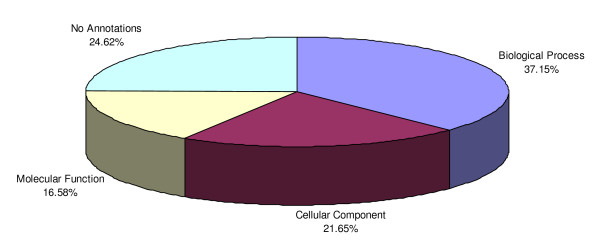
**Distribution of 3,594 genes by gene ontologies classification**. Genes with no annotation comprised 24.62% of the total, 37.15% were classified under the general category of biological process, 21.65% were classified according to their cellular component and 16.58% were classified based on their molecular function.

To gain insight of the genetic and biological events involved in the functional transition of the cotyledonary cell, we used the available gene ontologies (previously defined for the Blast X top hits) to classify under different functional categories the 3,594 genes with statistically significant data obtained in our study. Following the gene ontologies information, we first assigned the biological process classification associated with specific genes. This category includes biological processes comprising broad biological goals like metabolism, cell growth and cell death. Then genes were classified by cellular component which includes sub-cellular structures, locations and macromolecular complexes. Finally, genes were classified by molecular function which refers to the task performed by individual gene products [25,26]. For a total of 909 genes, a BlastX hit was produced along with the assigned hit description however the corresponding gene ontology information was not officially assigned at the time of the study. Therefore we assigned our own classification following the plant gene ontologies guidelines [27]. A summary of the classification of all the genes included in this collection is presented in Table 1. Interestingly 24.62% of genes with statistically significant data had no annotations either because the Blast X did not produce any hits below 10E-06, or because the hit was sub-classified into expressed, hypothetical, putative, unknown or unnamed protein; the rest of the genes were classified under biological process 37.15%, cellular component 21.65% and molecular function 16.58% (see Figure 3) (see Additional file 1: Complete collection of 3,594 genes with statistically significant data).

**Table 1:** Functional Classification of Genes with Statistically Significant Data

	**Total**	**Percentage**
**1. Biological Process**	**1335**	**37.15**
a. Cellular process		
i. Cell communication	1	0.03
ii. Cell Death	17	0.47
iii. Cell Differentiation	1	0.03
iv. Cell Growth and/or Maintenance	3	0.08
1. Cell growth	2	0.06
2. Transport	90	2.50
b. Physiological process		
i. Photosynthesis	11	0.31
ii. Response to stress	12	0.33
iii. Response to external stimulus	3	0.08
iv. Metabolism	373	10.38
1. Amino acid and derivative metabolism	14	0.39
2. Biosynthesis – Anabolism	43	1.20
3. Carbohydrate metabolism	74	2.06
4. Catabolism	22	0.61
5. Electron transport – transfer	91	2.53
6. Energy pathways	9	0.25
7. Lipid Metabolism	14	0.39
8. Nucleobase, nucleoside, nucleotide and nucleic acid metabolism	126	3.51
a. Transcription	41	1.14
9. Protein Metabolism	211	5.87
a. Protein Biosynthesis	120	3.34
b. Protein modification	7	0.19
10. Secondary metabolism	50	1.39
**2. Cellular Component**	**778**	**21.65**
a. Intracellular	12	0.33
i. Cytoplasm	2	0.06
1. Cytoskeleton	19	0.53
2. Endoplasmic Reticulum	3	0.08
3. Mitochondrion	148	4.12
4. Plastid Chloroplast	283	7.87
5. Ribosome	2	0.06
i. Nucleus	51	1.42
ii. Thylakoid	2	0.06
b. Membrane	233	6.48
i. Plasma Membrane, cell membrane, cytoplasmic membrane	23	0.64
**3. Molecular Function**	**596**	**16.58**
a. Catalytic activity	331	9.21
b. Binding	89	2.48
i. Nuclei acid binding	47	1.31
a. Transcription factor activity	78	2.17
b. Translation factor activity, nucleic acid binding	8	0.22
ii. Protein Binding	8	0.22
c. Molecular function unknown	10	0.28
d. Signal transducer activity	11	0.31
e. Structural molecule activity	8	0.22
f. Transporter activity	6	0.17
**4. No Annotations**	**885**	**24.62**

### Cluster analysis by *k-means*

**Figure 4:**
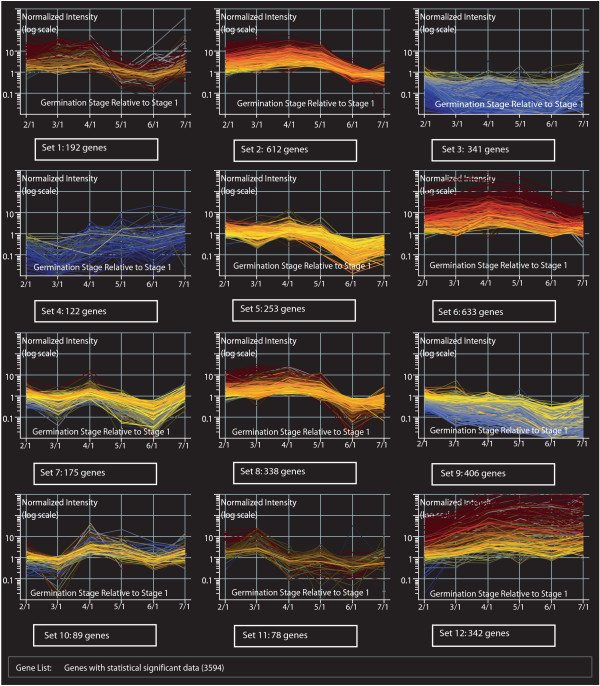
**Cluster analysis of genes with statistically significant data using *k-means***. Genes were clustered in a pre-determined number of 12 sets using the *k-means *algorithm available from Gene Spring 7.2 (Silicon Genetics, CA). Sets are numbered 1–12 and the total number of genes per set is recorded. Each set contains the schematic representation of the expression profile for each one of its members. On the X axis are the different germination stage (numerator) comparisons relative to the imbibed seed (denominator) in chronological order. The Y axis show the normalized intensity for each gene at each point on a log scale.

To better visualize the global gene transcription profile in the cotyledon during germination and to group genes that share similar profiles, we clustered the statistically significant genes into 12 sets according to their expression profiles throughout the different developmental stages using *k-means *(see Figure 4). This particular algorithm randomly divides genes into a user-defined number of clusters minimizing intra-cluster variability. Genes grouped within one set share a relatively similar expression profile however it is possible to find quantitative and qualitative expression differences among members of the same set. The more similar the expression profile is within a group of genes the more likely for those genes to be co-participants in the process of achieving a common biological function.

### Gene activity during functional transition of the cotyledon

Although each particular set obtained by cluster analysis represents a different group of genes with similar expression profiles, we can observe that sets 1, 2, 5, 8 and 11 contain genes whose expression profile displays up regulation during the first 3 to 4 stages, and variable levels of expression during the last 2 stages defined. The products of over-expressed genes during these initial stages may play a fundamental role in achieving the required functions of the cotyledon during the onset of seedling growth and specifically during the functional transition. Genes whose products participate in protein biosynthesis are especially abundant in this group (120 genes) (see Table 1, Additional file 1: Complete collection of 3,594 genes with statistically significant data) along with several transcription factors, regulators, elongation factors as well as ribosomal and aminoacid-metabolism genes. The data obtained clearly indicates high protein metabolism activity during the first stages of seedling development through the functional transition, and low activity during the later stages when the main function of these tissues is photosynthesis. Also a high number of genes were found within the subcategory of electron transport and protein and nuclei acid metabolism.

Close examination of the identified genes in these groups allowed us to define genes that have been previously associated with seedling development in different plant species. For instance, we found several chloroplast precursors like ferredoxin-NADP reductase root isozyme, several cytochrome P450 components and cystatin. Cystatins are ubiquitous plant cysteine peptidase inhibitors involved in the endogenous regulation of protein turnover, programmed cell death and in defence mechanisms [28]. Cystatins have been associated with germination in several plant systems like cowpea [29], barley [30], Chinese cabbage and transgenic *Arabidopsis *where they appear to participate in the control of seed germination by regulating cysteine peptidase activity [28]. Our data shows a steady over expression pattern during stages 2, 3 and 4 reduction during stage 5 and under-expression during stages 6 and 7.

In set 8, we can find genes like calnexin, ribonucleotide reductase R2, Kunitz trypsin inhibitor and putative bHLH transcription factor (see Table 2). The presence of transcripts from enzymes like the ribonucleotide reductase R2, and several DNA repair and DNA binding proteins agrees with the concept that DNA synthesis starts soon after seed imbibition [31] and is also complemented by the presence of multiple transcription factors like the putative bHLH transcription factor that has been reported to be the entry point for phytochrome regulation of the plant circadian clock and initiating one branch of the cytochrome-induced gene expression cascade [32]. Calnexin, is an endoplasmic reticulum trans-membrane molecular chaperone that selectively associates in a transient fashion with newly synthesized glycoproteins [33]. Expression of the calnexin is activated during late embryo development and germination in *Arabidopsis *(GER gene), showing the highest expression at 1 day after imbibition [34]. Our data shows that in soybean cotyledons, calnexin is over-expressed relative to the imbibed seed during the first 4 stages of germination with the highest peak at stage 4, to then become under-expressed during the last two stages (green tissues). The Kunitz trypsin inhibitor gene family contains at least 10 members, with qualitative and quantitative expression patterns programmed strictly by different and unique combinations of cis control elements per family member during the soybean life cycle [35]. These proteinase inhibitors function as storage proteins regulators of endogenous proteinases therefore playing an important role in regulating the mobilization of storage proteins during germination. We found that the Kunitz trypsin inhibitor in the soybean cotyledon cell was steadily up regulated during stages 2, 3, 4 and 5 while down regulated during stages 6 and 7. The corresponding EST clones to calnexin and Kunitz trypsin inhibitor [Clone ID: Gm-r1070-1638 and Gm-c1007-455] were used in an RNA blot hybridization experiment. The expression profiles obtained both by oligo microarray and RNA blot hybridization analysis were closely similar (data not shown).

**Table 2:** Selected Clones from 3,594 Genes with Statistically Significant Data

**Hit Description**	**Clone ID**	**Set^a^**	**2/1^b^**	**3/1^b^**	**4/1^b^**	**5/1^b^**	**6/1^b^**	**7/1^b^**
ABC transporter family protein	Gm-c1004-1877	4	ND	0.01*	0.34	0.45	0.29	0.41
ACTIN 3	Gm-r1021-1826	12	6.86	15.7	61.03	46.93	34.02	14.79
Acetyl-CoA C-acyltransferase precursor	Gm-r1089-1999	2	1.92	3.21	2.80	2.18	0.86	0.82
Aconitate hydratase, cytoplasmic	Gm-r1088-7889	2	6.76	13.11	11.72	3.38	0.36	0.41
Aconitate hydratase, cytoplasmic	Gm-r1088-8860	2	5.74	9.62	7.89	6.48	1.42	1.31
Aconitate hydratase, cytoplasmic	Gm-c1027-1520	8	11.91	2.86	5.20	3.11	0.11	0.49
ADR11^c^	Gm-r1021-2201	6	86.28	68.88	500.50	394.00	111.00	35.81
Alpha-carboxyltransferase precursor	U40979	6	10.3	22.23	43.74	31.66	34.69	8.00
Alpha' subunit of beta-conglycinin	Gm-r1070-250	9	0.33	0.48	0.37	0.12	0.04	0.11
Aspartate aminotransferase glyoxysomal isozyme	Gm-r1021-3097	8	1.91	2.88	2.48	1.04	0.21	0.28
Aux/IAA protein	Gm-r1021-1791	12	ND	9.3	25.1	13.98	48.07	14.62
Auxin down regulated ADR12-2^d^	Gm-r1083-898	10	0.15	0.12	8.92	2.80	4.75	2.24
Auxin down regulated ADR6^c^	Gm-r1021-3058	12	20.27	62.24	65.15	124.60	244.60	424.00
Auxin down regulated ADS11-2^c^	Gm-c1045-5622	12	22.00	36.49	42.67	86.56	118.70	11.32
Auxin transport protein (BIG)	Gm-c1062-1281	1	2.71	22.68	8.69	0.33	0.15	0.77
Auxin-binding protein ABP19	Gm-r1083-1259	12	15.01	47.63	343.00	364.50	161.80	61.94
Auxin-induced protein 22C	Gm-c1031-62	12	0.98	7.15	11.41	14.73	15.68	30.11
Beta-amylase^c^	Gm-c1016-12242	4	0.02	0.01	0.36	5.75	8.39	6.11
Beta-conglycinin alpha-subunit^c^	Gm-c1007-354	4	0.46	0.06	0.06	0.27	3.95	2.51
Beta-tubulin	Gm-r1021-2933	12	0.89	3.96	23.40	39.75	18.31	2.58
Beta-tubulin	Gm-c1063-3619	2	4.77	3.74	7.31	3.51	0.70	0.99
Calnexin^c^	Gm-r1070-1638	8	2.12	2.23	4.88	1.64	0.34	0.36
Catalase	Gm-c1027-5248	6	3.36	3.22	4.13	3.06	1.13	2.15
Catalase	Gm-r1088-8229	6	3.25	3.63	4.91	3.57	1.41	2.17
Cell division cycle protein 48 homolog	Gm-c1019-5885	8	1.17	1.40	1.67	1.49	0.47	0.44
Cellulose synthase	Gm-c1046-518	1	0.77	4.01	11.08	0.53	0.37	1.40
Chalcone isomerase 1A	Gm-c1065-8978	5	2.04	2.29	2.47	0.59	0.18	0.35
Chalcone synthase CHS1	Gm-r1083-1425	5	1.05	0.69	0.82	0.61	0.07	0.17
Chlorophyll a/b binding protein^d^	Gm-r1083-1883	12	16.02	38.60	338.10	228.60	930.50	543.60
Chlorophyll a/b binding protein CP29	Gm-r1083-1764	12	7.55	16.05	122.70	147.30	131.10	83.41
Chloroplast ATP synthase (delta subunit)	Gm-r1021-3478	12	4.35	19.10	52.30	28.10	22.19	22.04
Chloroplast inner membrane import protein Tic22	Gm-r1070-7629	8	2.25	3.16	4.81	2.29	0.33	0.79
Chloroplast outer envelope protein 34	Gm-c1004-4823	8	2.74	1.70	3.54	5.35	0.16	0.17
Chloroplastic outer envelope membrane protein	Gm-r1089-5811	8	9.36	16.07	7.24	10.44	0.19	0.48
Citrate synthase, glyoxysomal precursor	Gm-r1070-1609	5	1.17	2.66	1.89	0.72	0.15	0.44
Citrate synthase	Gm-c1084-1995	8	1.28	1.68	2.04	1.18	0.44	0.43
Citrate synthase	Gm-r1070-5048	2	2.49	3.10	4.59	2.95	0.97	0.46
Citrate synthase	Gm-r1070-5826	2	1.93	2.44	4.09	2.36	0.80	0.51
Citrate synthase	Gm-c1028-1504	8	17.9	18.2	5.11	0.9	ND	0.28
Clathrin coat assembly like protein^c^	Gm-r1083-3129	6	1.16	1.40	11.83	5.69	1.41	1.39
Coproporphyrinogen oxidase precursor	Gm-r1070-7634	8	1.58	1.57	2.24	1.89	0.60	0.35
Cystatin	Gm-r1088-2533	5	2.60	2.04	2.39	1.20	0.11	0.03
Cytochrome c1 precursor	Gm-c1044-29	6	2.97	2.21	12.37	11.96	1.23	1.85
Cytochrome b5	Gm-r1021-516	6	0.56	2.17	8.70	6.33	2.91	0.68
Cytochrome b5 reductase	Gm-r1021-1680	6	21.1	8.30	39.74	15.86	3.92	2.47
Cytochrome c1 precursor	Gm-c1044-29	6	2.97	2.21	12.37	11.96	1.24	1.86
Cytochrome oxidase subunit 2	Gm-c1013-3829	6	2.78	2.18	5.68	6.95	1.27	2.21
Cytochrome P450	Gm-r1083-2584	6	14.20	14.62	29.92	9.55	2.59	12.03
Cytosolic malate dehydrogenase	Gm-c1048-4886	6	22.21	7.55	67.02	67.17	7.15	10.83
Dehydration responsive element binding protein	Gm-r1088-7881	9	0.75	1.36	0.50	0.23	0.06	0.45
Elongation factor 1-alpha	Gm-r1089-3525	2	2.62	1.41	3.62	1.93	0.68	0.82
Expansin	Gm-c1040-1709	8	1.54	2.19	3.21	2.63	0.57	0.51
Ferredoxin – NADP reductase, chloroplast prec	Gm-c1077-506	5	1.12	2.50	1.09	1.04	0.16	0.20
Formate dehydrogenase	Gm-r1021-2716	12	1.35	1.35	7.48	4.81	2.81	4.93
Glutamate aminotransferase	Gm-c1065-9456	6	22.37	37.99	67.99	9.41	9.57	1.96
Glutathione S-transferase GST 10	Gm-r1083-1120	9	0.94	0.81	0.64	0.36	0.08	0.11
Glycolate oxidase	Gm-c1020-110	12	12.74	19.52	48.55	78.47	84.34	58.77
Histone H1, drought-inducible	Gm-r1021-1206	9	1.13	0.26	0.38	0.20	0.06	0.28
Isocitrate lyase, ICL 1	L02329	6	112.6	407.5	342.7	207.6	24.28	35.43
Isocitrate lyase, ICL 2	Gm-r1088-3791	6	153.1	462.0	325.9	212.9	36.24	29.45
Kunitz trypsin inhibitor^c^	Gm-c1007-455	8	4.66	10.61	8.00	5.25	0.13	0.34
Late-embryogenesis abundant protein	Gm-r1089-1615	9	0.54	0.63	0.55	0.41	0.03	0.08
LHCII type III chlorophyll a/b binding protein	Gm-r1083-1883	12	4.70	40.17	327.00	684.00	1322.00	693.70
Lipoxygenase L-5	Gm-c1071-6455	12	69.06	107.30	835.10	2362.00	789.20	1101.00
Long-chain acyl-CoA synthetase	Gm-r1070-2169	8	1.67	2.59	1.86	1.37	0.40	0.94
Malate dehydrogenase, glyoxysomal precursor	Gm-r1088-2531	2	6.44	11.18	10.50	3.20	0.58	0.81
Malate dehydrogenase, chloroplast	Gm-r1070-1784	8	1.17	1.66	3.46	1.86	0.27	0.37
Malate dehydrogenase, mitochondria	Gm-r1021-57	6	7.96	1.93	23.12	10.29	1.82	4.12
Malate dehydrogenase, mitochondria	AF068689	6	5.39	5.51	9.42	6.71	1.30	2.49
Malate dehydrogenase, ATP binding	Gm-r1088-7885	9	0.19	0.15	0.05	0.03	0.01	0.02
Malate synthase, glyoxysomal	Gm-r1070-7761	2	3.62	4.86	2.78	2.18	0.46	0.81
Malate synthase, glyoxysomal	Gm-r1088-2873	8	2.38	2.56	4.03	2.32	0.41	0.47
Malate synthase, glyoxysomal	Gm-r1070-8044	8	4.24	5.48	5.85	2.63	0.23	0.60
NADPH-specific isocitrate dehydrogenase	Gm-r1021-3472	8	1.77	1.72	2.77	1.74	0.33	0.83
Oxalyl-CoA decarboxylase	Gm-r1089-8230	7	1.03	0.55	1.43	0.07	0.22	1.47
Oxoglutarate malate translocator	Gm-c1062-4128	6	7.38	17.38	15.24	11.81	3.38	2.06
Peroxiredoxin	Gm-r1021-6	6	6.87	9.76	22.85	22.03	13.72	4.03
Phosphoglycerate kinase	Gm-r1083-1861	12	2.97	1.89	4.54	26.45	16.3	3.17
Phytochrome A	Gm-r1089-5497	6	10.09	16.54	21.62	8.51	5.55	3.35
Polygalacturonase	Gm-r1021-1072	6	25.88	14.38	52.60	31.02	38.59	4.09
Probable lipid transfer protein M30 precursor	Gm-r1089-5540	6	9.71	8.64	12.78	14.57	3.87	1.83
Probable mitotic control protein dis3	Gm-r1070-9203	8	1.49	3.94	2.18	2.15	0.40	0.89
Putative bHLH transcription factor	Gm-c1028-1591	8	6.98	9.76	5.59	1.03	0.40	0.43
Putative cell division related protein	Gm-r1083-1257	8	1.29	2.29	3.22	0.61	0.58	0.35
Putative lipase	Gm-c1013-3043	6	6.73	3.52	16.43	17.29	4.92	2.16
Putative protein phosphatase-2C	Gm-r1021-519	4	0.70	0.26	0.11	1.72	2.20	2.26
Ribonucleotide reductase R2	Gm-r1083-349	8	3.29	12.00	15.27	3.04	0.13	0.22
RUB1 conjugating enzyme	Gm-r1088-5674	6	1.62	1.85	3.17	2.09	1.26	2.42
Rubisco	Gm-c1069-8064	12	106.5	170.0	2,085	1,324	1,478	1,617
Rubisco small chain precursor	Gm-c1047-446	12	51.70	149.40	1210.00	1110.00	1489.00	861.40
RuBisCO-associated protein	Gm-r1021-1539	6	2.62	1.86	16.54	3.32	2.21	0.93
Seed maturation protein LEA 4	Gm-c1068-7258	9	0.20	0.14	0.11	0.06	0.02	0.03
Transcription factor BTF3	Gm-r1021-739	6	3.31	6.98	10.87	6.03	2.15	1.51
Transcription factor EIL2	Gm-c1020-311	1	5.01	2.78	7.41	0.62	2.44	2.44
Transcription factor lim1	Gm-r1088-3755	6	10.85	16.00	35.32	27.99	8.34	5.18
Transcription factor RAU1	Gm-r1088-6520	6	8.82	12.84	21.78	5.62	2.85	13.14
Transcription regulator protein SNF2	Gm-c1064-4573	6	3.02	13.31	3.76	4.00	1.30	2.18
Transcription repressor ROM1	Gm-r1088-612	6	1.14	3.33	3.53	3.42	1.08	2.19
Tubulin a-1 chain	Gm-r1021-3527	6	1.30	1.97	6.37	6.54	1.39	1.24
Ubiquinol-cytochrome C reductase	Gm-r1070-5286	6	3.19	4.62	5.89	7.84	1.73	1.37
Unknown^c^	Gm-c1044-260	4	0.51	ND	0.148	0.404	1.353	2.419
Unknown^c^	Gm-c1032-1982	8	4.66	10.54	7.552	1.495	0.169	0.675
Unknown^c^	Gm-r1070-6924	2	1.96	2.571	14.35	7.514	0.778	0.773

^a ^Set number assigned from cluster analysis by *k-means*

^b ^X/1 Indicates expression ratio of specific stage X (2–7) to stage 1 (reference tissue). n = 8

^c ^Clones validated by RNA gel blot analysis

^d ^Clones validated by RNA gel blot analysis and qRTPCR

Some other gene expression profiles initially show activity during the stages of the functional transition and continue to be active throughout the green stages. Genes with this particular profile are more likely to participate in the different physiological processes relevant to these developmental stages. Some of the genes found within this set are: β-amylase, putative protein phosphatase-2C, β-conglycinin alpha subunit, glucosyltransferase-5, ABC transporter family members, and seed maturation proteins PM34 and PM31.

The β-amylase gene has been previously characterized in maize in association with seed germination where high expression was observed after day 2 reaching peaks at days 5–6 and maintained high levels through days 7–10 [36]. Our data shows that the soybean β-amylase gene in the cotyledon (see Table 2) is down regulated during stages 2, 3 and 4, and up regulated during stages 5, 6 and 7. The β-conglycinin is one of the major seed storage globulins in soybean. The 3 subunits of β-conglycinin (α, α' and β) are rapidly degraded after seed imbibition generating new β-conglycinin cross-reactive polypeptides. After six days of germination the newly produced β-conglycinin polypeptides are also hydrolyzed [37]. These observations are supported by our data in that the gene for β-conglycinin shows low levels of expression (see Table 2) during the initial stages of germination (when degradation of β-conglycinin subunits is occurring) and an increase in expression during stages 6 and 7 (photosynthetic stages). It has been postulated [38] that the newly produced β-conglycinin polypeptides may play a role in the photosynthetic activity of the cotyledon during the late stages of germination. The corresponding EST clones to β-amylase and β-conglycinin [Clone ID: Gm-c1016-12242 and Gm-c1007-354] were selected, purified and used as radio-labeled probes in an RNA blot that contained total RNA from the imbibed seed (reference tissue) and the remaining six different stages from one of the biological replicates previously used. These RNA blots produced similar expression profiles to the ones observed by oligo microarray analysis (data not shown).

### Specific elements of the glyoxylate pathway involved in the functional transition

**Figure 5:**
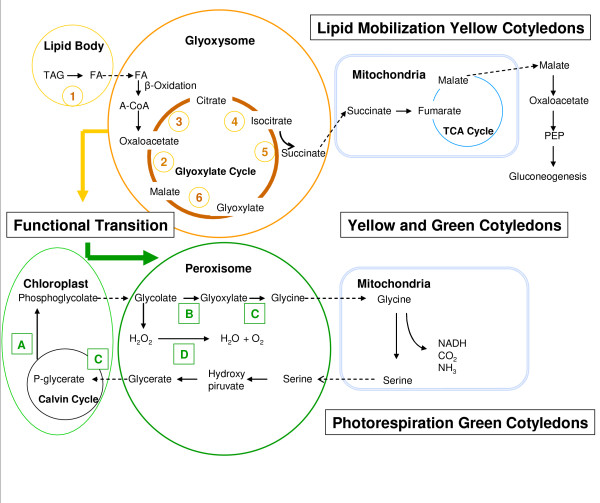
**Lipid mobilization and photorespiration processes in the soybean cotyledon cell during functional transition**. During the first stages of seedling development, when lipid mobilization is essential, lipase (1) catalyses the release of fatty acids (FA) from triacylglycerol (TAG) within the lipid bodies. The fatty acids produced are activated to acyl-CoA esters and enter the glyoxysome where the acyl-CoA molecules are broken down to acetyl-CoA via the β-oxidation spiral. The acetyl-CoA then serves as a substrate for the subsequent reactions of the glyoxylate cycle which is an adapted form of the respiratory tricarboxylic acid (TCA) cycle. Succinate is produced, transformed to malate in the mitochondrion and exported to the cytoplasm. Then, it is converted to carbohydrate which is translocated and used in the growing parts of the plant. Glyoxysomes and peroxisomes co-exist during the functional transition and eventually peroxisomes become abundant while glyoxysomes decrease in number. During the photorespiratory phase the phosphoglycolate produced in the chloroplast through the Calvin cycle is mobilized to the peroxisome in the form of glycolate where is oxidized to glyoxylate in a reaction that yields hydrogen peroxide. Glycine is finally synthesized and transferred to the mitochondrion, converted to serine and returned to the chloroplast in the form of glycerate after several metabolic reactions within the peroxisome. Peroxisomes are commonly large and abundant in photosynthetic tissues. 1 – Lipase; 2 – Malate dehydrogenase (MDH); 3 – Citrate synthase (CS); 4 – Aconitate hydratase (AH); 5 – Isocitrate lyase (ICL); 6 – Malate synthase (MS); A – Ribulose-bisphosphate caroboxylase (Rubisco); B – Glycolate oxidase; C – glutamate semialdehyde aminotransferase; D – Catalase; E – phosphoglycerate kinase.

**Figure 6:**
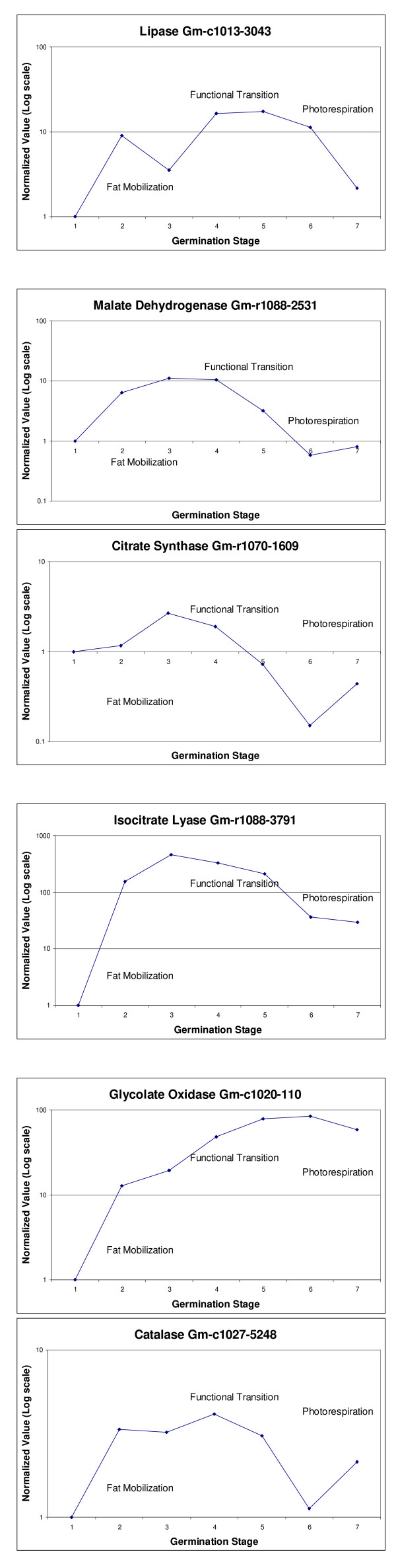
**Oligo microarray expression profiles during soybean seedling development of selected clones**. Expression profiles throughout seedling development show the normalized ratio (log scale) of every stage measured relative to stage 1 (imbibed seed). Stages 2 and 3 are characterized by fat mobilization, stages 3 and 4 represent the functional transition when the cotyledons are green and yellow and stages 6 and 7 represent the green photosynthetic tissues. A value of 1 indicates no change in expression relative to the imbibed seed. Values >1 indicate up regulation while values <1 indicate down regulation.

An interesting group of genes found with an expression profile that shows to be relevant during soybean seedling development is a set of genes that directly or indirectly participate in the glyoxylate cycle. The glyoxylate cycle has been associated with the mobilization and utilization of lipids during germination in several plant species characterized by oil-rich seeds [12-14]. Initially lipases in oil bodies start the process of fat utilization by carrying out the hydrolytic fission of triacylglycerol (TAG) to free fatty acids and glycerol (see Figure 5). In castor bean two lipases have been identified, one is acidic and accumulates during the first two days of germination and the other one is alkaline accumulating during days 3–5 [38]. We found six different genes that codify for lipases but only one specific lipase gene [Clone ID: Gm-c1013-3043] that has been associated with TAG lipase activity. The expression profile of this gene fluctuates between high levels of expression during the initial phases of germination reaching higher peaks during the functional transition and a significant reduction of expression during the last two stages defined (see Figure 6). The other lipases found in our study are mitochondrial, plastid or associated with the endomembrane system. Lipoxygenase has been proposed to be involved in reserve lipid mobilization during soybean germination and although high levels have been detected during this period, recent studies suggest that lipoxygenase is not directly involved in lipid mobilization and its function is still unclear [39]. We found five different lipoxygenase genes [Clone ID: Gm-r1089-2163, Gm-c1071-6455, GenBank Accesions: U36191, U50081 and J03211] with similar expression profiles and higher levels of expression during the functional transitions (stages 4 and 5). We also found a lipoxygenase gene whose expression profile showed down regulation during the entire study.

Our data shows that specific genes codifying for enzymes from the glyoxylate pathway like malate dehydrogenase (MDH), citrate synthase (CS), aconitate hydratase (AH), isocitrate lyase (ICL), malate synthase (MS), formate dehydrogenase (FDH), oxalyl-CoA decarboxylase (OCD) and glyoxylate carboligase (GXC) are up regulated at some point during the first four stages of soybean seedling development and down regulated during the last two stages defined (see Figure 5) correlating with the cotyledonary functional transition.

The malate dehydrogenase (MDH) is one of the key enzymes in the glyoxylate cycle in charge of converting malate to oxaloacetate (see Figure 5) and has been associated with elevated expression during germination in oilseed plants [12]. We found in our collection of genes with statistical significant data (3,594) (see Additional file 1: Complete collection of 3,594 genes with statistically significant data), six different MDH genes with different expression profiles: one is cytosolic [Clone ID: Gm-c1048-4886], two are mitochondrial [Clone ID: Gm-r1021-57 and GenBank Accesion: AF068689], one is glyoxysomal [Clone ID:Gm-r1088-2531], one is from chloroplast [Gm-r1070-1784] and one is registered as an ATP binding MDH [Gm-r1088-7885]. Due to the nature of the soybean oligo array we can clearly distinguish the individual expression profiles of each one of these genes and define the specific isoform that has a relevant role at a particular point during our study. Out of the six MDH genes found, only the ATP binding MDH gene [Clone ID:Gm-r1088-7885] was consistently down regulated throughout the study, implying that this particular isoform might not be as relevant during early seedling development, while the other five MDH genes reached peaks of high levels of expression during the functional transition stages. The cytosolic MDH gene [Clone ID: Gm-c1048-4886], is up regulated in all of the stages defined and presents the highest levels of expression among all the six found during the cotyledonary functional transition (stages 4 and 5). The glyoxysomal MDH gene [Clone ID: Gm-r1088-2531], is up regulated during the first stages with higher peaks at stages 3 and 4. At stage 5 the expression levels of this particular MDH are reduced by about 50% relative to stage 4. A significant reduction in levels of expression was observed during the green stages of cotyledon development during seedling development (see Figure 6). The expression profiles of the two mitochondrial isoform genes are very similar, showing up regulation during the first 4 stages (2 to 5) and a significant reduction during the last two (green tissues). However, the reduced levels of expression in the green stages were still higher than the ones observed in the imbibed seed. The mitochondrial MDH is associated with the TCA Cycle. The chloroplast MDH gene [Clone ID: Gm-r1070-1784], showed a steady increase in expression from imbibition to stage 4, a slight reduction during stage 5 and down regulation during stages 6 and 7.

Citrate synthase (CS) also named as citrate lyase, is one of the key enzymes of the glyoxylate cycle (see Figure 5) in charge of producing citrate from acetyl CoA and oxaloacetate [13,15]. In our study we found five genes [Clone ID: Gm-r1070-1609, Gm-c1084-1995, Gm-r1070-5048, Gm-r1070-5826, Gm-c1028-1504] with elevated levels of expression during the initial phases of germination and low levels during the photosynthetic phase. Only one of these genes [Clone ID: Gm-r1070-1609] is referenced as a glyoxysomal precursor showing up regulation during stages 3 and 4 and different levels of down regulation in the rest of the stages defined (see Figure 6). We were unable to identify the CS related with the TCA cycle in the mitochondrion.

Aconitate hydratase (AH) is in charge of producing isocitrate from citrate (see Figure 5). Two different AH enzymes have been associated with the process of germination in several oil-seed plants. One is mitochondrial and involved the TCA cycle while the other one is cytosolic participating in the glyoxylate cycle [40]. There are three different genes [Clone ID: Gm-c1027-1520, Gm-r1088-7889, Gm-r1088-8860] with statistically significant data that codify for AH in our study. As with some of the other enzymes from the glyoxylate cycle found in this study, the AH expression profiles of these three genes in the soybean cotyledon start with up regulation during the first stages of germination and down regulation after the functional transition during the green phases.

Isocitrate lyase (ICL) produces succinate and glyoxylate from isocitrate (see Figure 5). It has been used in immunological assays to demonstrate the inter-conversion of peroxisomes to glyoxysomes and vice versa in pumpkin cotyledons during germination and senescence [41]. Increased levels of expression have been documented during germination in glyoxysomes while low levels of expression have been observed in peroxisomes during cotyledonary greening and senescence [41]. We found two ICL genes (ICL 1, GenBank Accesion: L02329; ICL2, Clone ID: Gm-r1088-3791) showing almost identical expression profiles. Both genes show up regulation during the entire time study however, reduced levels of expression in one order of magnitude were observed after completion of the cotyledonary functional transition (see Figure 6).

Malate Synthase (MS) activity have been shown to increase co-ordinately (along with other enzymes from the glyoxylate and TCA cycle) with patterns of lipid breakdown (see Figure 5) during germination in different plant species, and has been used as a marker for glyoxysomal activity [42,43]. We found three different glyoxysomal MS isoform genes [Clone ID: Gm-r1070-7761, Gm-r1070-8044, Gm-r1088-2873] with similar expression profiles that start with up regulation during the initial phases of germination, down regulation right after the cotyledonary functional transition and during the photosynthetic phase (see Table 2).

Several other enzymes related with the glyoxylate cycle are FDH, OCD and GXC. We found one specific element for each of these enzymes in our collection of genes with statistically significant data: Clone ID: Gm-r1021-2716 for FDH; Clone ID: Gm-r1089-8230 for OCD and Clone ID: Gm-c1062-4128 for GXC. The gene for FDH shows low levels of expression relative to the imbibed seed during the first stages of seedling development, high levels of expression at the onset of the functional transition and during the photosynthetic phase (green tissues). The expression profile of the gene for OCD on the other hand shows down regulation throughout the entire study indicating that this particular branch of the cycle may not be required in the soybean cotyledon during the functional transition in the process of seedling development. Finally we found that the expression profile of the GXC gene our study is up regulated in all stages with a reduction of expression during the green phase (stages 6 and 7).

We also observed transcript accumulation from genes that are involved in peroxisomal activity as glycolate oxidase [Clone ID: Gm-c1020-110] (see Figure 5 and 6), glutamate aminotransferase [Clone ID: Gm-c1065-9456] and catalase [Clone ID: Gm-c1027-5248, Gm-r1088-8229] (see Figure 5 and 6) along with others that participate in the process of photorespiration like Rubisco [Clone ID: Gm-c1069-8064] and phosphoglycerate kinase [Clone ID: Gm-r1083-1861]. The expression profiles of these genes show different levels of up regulation throughout most of the stages defined indicating peroxisomal activity in the soybean cotyledon during seedling development (see Figure 5).

### Highly active genes throughout seedling development

On the other hand, consistently over expressed genes throughout the entire time study in relation to the imbibed seed are mainly found in sets 6 and 12 from cluster analysis. These are genes whose expression was reduced in the imbibed seed and increased with the onset of germination, remaining over-expressed throughout the study. Within set 6, metabolism of nuclei acids, electron transport and protein biosynthesis are the functional sub-classes with higher number of genes (see Table 1). Several transcription factors can be found within this group like: transcription factor homolog BTF3-like protein, transcription factor lim1, transcription factor RAU1, transcription regulatory protein SNF2 and transcription repressor ROM1 (see Table 2). Also genes involved in lipid metabolism like probable lipid transfer protein M30 precursor, peroxiredoxin, polygalacturonase beta chain precursor, alfa-carboxyltransferase precursor, tubulin a-1 and b chain, and clathrin coat assembly like protein are present within this group. Similarly several photosynthetic genes like RUB1 conjugating enzyme and RuBisCO-associated protein, cytochromes (cytochrome oxidase subunit 2, cytochrome b5, cytochrome P450, ubiquinol-cytochrome C reductase complex, cytochrome c1 precursor, cytochrome-b5 reductase-like protein) and phytochrome A, can also be found within this set of genes.

Some of these gene products have been associated with the process of germination in different species and for some of them their functions have been studied. For example, peroxiredoxins are thiol-requiring antioxidants that serve as general cellular reductants that are tightly regulated during seed development, seed imbibition and early germination [44]. Peroxiredoxins may play a role in protecting the growing embryo from reactive oxygen species arising as by products of respiration during imbibition and germination [45]. We identified one peroxiredoxin gene [Clone ID: Gm-r1021-6] in the soybean cotyledon that shows over-expression throughout our study with particularly high peaks during stages 4 and 5 (functional transition). In tomato, polygalacturonase is believed to play an important function in weakening of the endosperm tissue opposite to the radical tip allowing radicle protrusion; mRNA abundance is low during seed development, increased during imbibition and highly abundant in seeds that have completed germination [46]. Our data suggests that the gene for polygalacturonase [Clone ID: Gm-r1021-1072] in soybean cotyledons is up regulated during seedling development with the highest peak at stage 4 and the lowest at the end of this process (stage 7). We also found several members of the tubulin gene family within this group and particularly one [Clone ID: Gm-r1021-3527] with higher levels of expression during the functional transition. Tubulins have been associated with cell division and enlargement aspects of the cell cycle accumulating during early stages of germination depending on the action of giberellic acid in *Arabidopsis *[47]. Clathrin coated vesicles (CV) play an important role in transport of storage proteins during seed development, and appeared to be enriched in pea cotyledons during germination [48]. Our data shows high expression of the clathrin coat assembly like gene [Clone ID: Gm-r1083-3129] during stages 4 and 5 (functional transition) indicating possible accumulation of CV's that are involved in protein transport.

Set 12 is characterized by genes that were highly over-expressed throughout the entire time study. Some relevant genes found within this group are: ACTIN 3, aux/IAA protein, auxin down regulated ADR6 and ADS 11-2, auxin-binding protein ABP19, auxin-induced protein 22C, auxin-induced protein aux22 and aux28, chlorophyll a/b-binding protein (CP29, type II, LHCII type III and type I precursor), chloroplast ATP synthase, plastocyanin precursor, beta-tubulin, and Rubisco small chain precursor.

**Figure 7:**
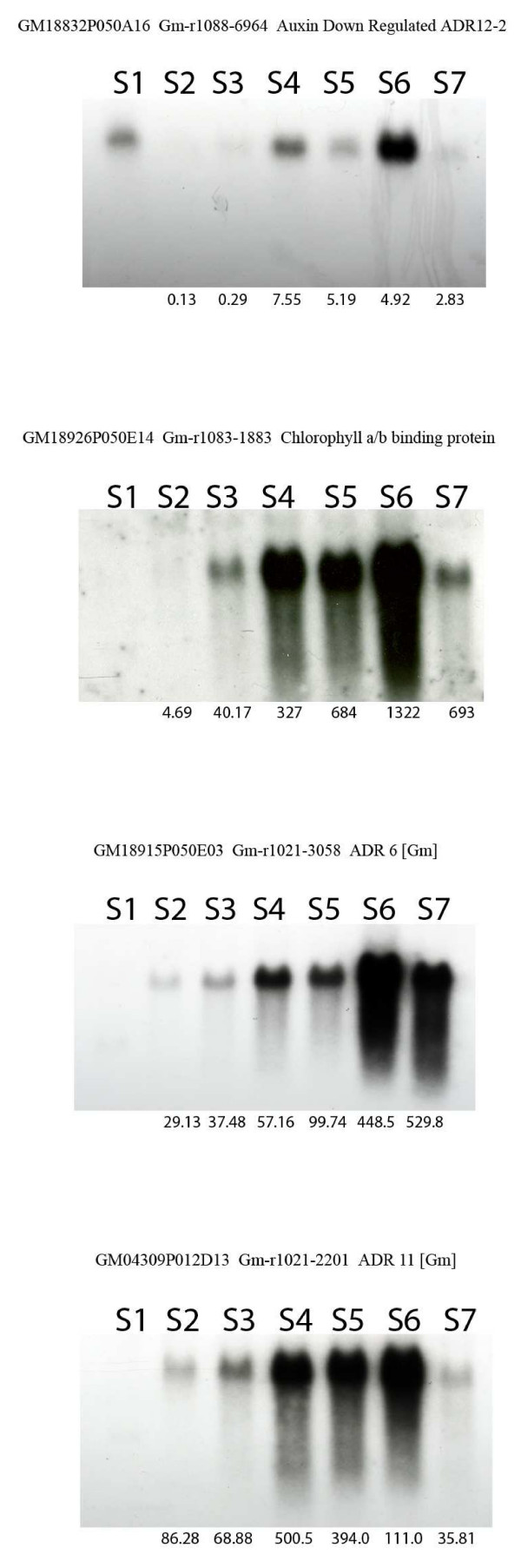
**RNA gel blot hybridization analysis of four selected genes**. Total RNA (10 μg) from cotyledons of the reference tissue (S1) and the subsequent stages (S2–S7) was prepared in 6.5% formaldehyde and 50% formamide, electrophoresed on a 1.2% agarose, 1.1% formaldehyde gel and blotted onto a nitrocellulose membrane [61]. The blots were hybridized to radioactive probes obtained from the soybean public EST collection that contained the specific oligo sequence to be validated. These clones were hand picked, grown, purified, confirmed by sequence analysis and radioactively labeled. The relative values obtained by microarray data analysis are reported at the bottom of each lane.

**Figure 8:**
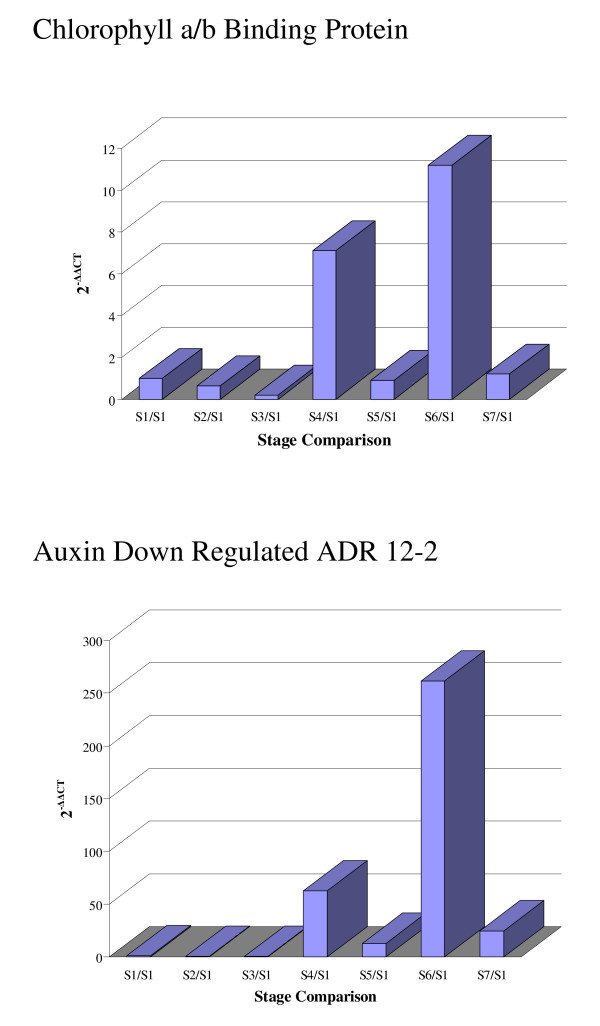
**Microarray data validation by qRTPCR**. Specific primers were designed to amplify exclusively the oligo sequences found by microarray data and confirmed by RNA gel blot analysis. The two clones chosen were the Auxin down regulated ADR12-2 and the Chlorophyll a/b binding protein. All reactions were conducted in triplicate starting with purified total RNA (5 μg) from each developmental stage (S1–S7) from one biological replicate that was converted into first strand cDNA by reverse transcription. PCR product quantification was based on a Ct value. The relative expression of each stage (S2–S7) compared to the reference imbibed seed tissue (S1) was calculated by averaging the 3 Ct values for each measurement and determine the relative ratio using the 2^-ΔΔCt ^method [62].

Chorophyll a/b binding (Cab) proteins are synthesized in the cytoplasm and transported into the chloroplast where they bind chlorophyll a and b and carotenoid pigments to form pigment/protein complexes. These complexes transfer light energy to photosystem reaction centres and mediate thylakoid membrane appression. Cab mRNA levels have been reported to fluctuate in the soybean embryo during seed development and maturation as well as in the cotyledons during germination; their expression is negatively regulated by abscisic acid [49]. Our data shows up regulation of several Cab genes within the cotyledon cell with increasing levels from stage 2 to 6 and then a small reduction at stage 7. One of the corresponding EST clone for Cab [Clone ID: Gm-r1083-1883] was used as a probe in an RNA blot analysis containing total RNA samples from all the stages in one of the biological replicates. A similar profile compared with the one from the oligo microarray analysis was obtained (see Figure 7). Additionally, specific primers flanking the 70-mer oligo used in the array were designed and used in a qRT-PCR reaction (see Methods) starting with total RNA from the same biological replicate used for the RNA blot hybridization analysis. The results from the qRT-PCR validated both the oligo microarrray data as well as the northern data (see Figure 8).

The phytohormone auxin down regulates the expression of a group of genes known as the auxin down regulated (ADR) genes of which there are distinctly different families by genomic sequence. ADR genes also respond to light differentially and are organ/tissue specific. ADR 6 and ADR 11 mRNA cytoplasmic levels are low in the presence of auxin, while in the presence of light ADR 6 is up regulated and ADR 11 is down-regulated [50]. The majority of the ADR genes in our study were grouped in set 12 (steady up-regulation), however the ADR 12-2 profile was classified in set 10 showing under-expression during stages 2 and 3 and different levels of over-expression during stages 4 through 7 (see Figure 7). The corresponding EST clones for auxin down regulated ADR 6, ADR 11 and ADR 12-2 [Clone ID: Gm-r1021-3058, Gm-c1021-2201 and Gm-r1088-6964 respectively] were purified, sequenced and used as radio-labeled probes in an RNA blot that contained total RNA from the imbibed seed (stage1, reference tissue) and the remaining six different stages (2–7) from one of the biological replicates previously used. The results showed similar expression profiles to the corresponding ones observed by oligo microarray analysis (see Figure 7). In addition, specific primers flanking the 70-mer oligo used in the array for the ADR12-2 gene were designed and used in a qRT-PCR reaction (see Methods) starting with total RNA from the same biological replicate used for the RNA blot hybridization analysis. The results from the qRT-PCR validated the data obtained by the other two independent methods (see Figure 8).

### Down regulated genes throughout seedling development

Genes that were under-expressed during the entire time study are those genes that were up-regulated in the imbibed seed (reference tissue) and their expression was reduced throughout all the subsequent stages during seedling development. These genes are mainly found in sets 3 and 9 (see Additional file 1: Complete collection of 3,594 genes with statistically significant data).

Some of the genes found in set 9 are: wound induced protein, late-embryogenesis abundant protein, glutathione S-transferase GST 6, 7, 10, 11, 13, 14, 15 and 17, histone H1 (drought-inducible) and H3.2, Alpha' subunit of beta-conglycinin, dehydration responsive element binding protein, seed maturation proteins PM40, 37, 29 and 30, soybean seed maturation polypeptides, and several members of the flavonoid pathway like chalcone reductase, chalcone synthase CHS 3, 4, 7 and 8, cinnamate 4-hydroxylase, naringenin-chalcone synthase and isoflavone reductase homolog 1. In soybean isoflavone genes are expressed at different levels throughout development in tissues such as seeds and roots, while in other tissues they are further induced in response to elicitor treatment or pathogen attack [51,52]. Accumulation of isoflavone transcripts has been also associated with cotyledon development during seed maturation [53] as well as tissue-culture induced somatic embryo development [21]. Our data shows that during early seedling development accumulation of isoflavone transcripts is reduced in relation to the imbibed seed cotyledons. Finally, the seed maturation proteins are synthesized during the late stage of seed maturation and have been correlated with desiccation tolerance, ABA content or transition to seedling growth; they tend to highly accumulate in dry and mature soybean seeds degrading rapidly at the early stage of seed germination [54].

### Data validation by RNA blot hybridization analysis and qRT-PCR

Based on the oligo microarray data obtained in our study along with literature evidence of involvement in the process of early seedling development in plants, several gene candidates were chosen (see Table 2, Figure 7) to confirm their transcript abundance profile throughout the six developmental time points defined in comparison to the imbibed seed (see Figure 1) by RNA blot hybridization analysis. Unknown genes were also selected based on their similarity to the expression profiles of the previously chosen genes. For this purpose individual EST clones corresponding to the oligo sequence used in the microarray study were hand picked form the EST collection available, purified, confirmed by sequence analysis, radioactively labeled and used as probes on an RNA blot containing purified total RNA from the seven stages of one biological replicate used for this study (see Methods). Figure 7, shows results of the RNA gel blot hybridization using the chlorophyll a/b binding protein, auxin down regulated ADR 12-2, ADR 6 and ADR 11 probes along with the values and expression profile obtained from the oligo microarray data. Similar data was obtained for the rest of the clones registered in Table 1 (data not shown).

For qRT-PCR, primers were designed to amplify exclusively the oligo sequences found by microarray data and confirmed by RNA gel blot analysis. The two clones chosen were the auxin down regulated ADR12-2 and the chlorophyll a/b binding protein. The qRT-PCR results for these two genes indicated that transcript abundance profiles were consistent with the oligo microarray results as well as the RNA gel blot hybridizations (see Figure 8).

## Discussion

A soybean 70-mer oligo array containing 19,200 features was used to study the modulation of the global transcript profile of soybean cotyledons at 6 different time points relative to the imbibed seed. Our time study covered the cotyledon functional transition from nutrient storage to photosynthesis during seedling development. One of the major advantages of using an oligo array of this nature is that it allows to clearly discriminate the expression levels in terms of transcript abundance of different gene family members, enzymatic isoforms and protein subunits. This is due to the fact that each 70-mer oligo has been carefully designed to represent the high sequence variability found at the most possible 3'end region of cDNA clones of the soybean EST collection. The results of our study define which specific gene family member, enzymatic isoform or protein subunit is likely to be a co-participant of this particular biological process providing an important tool, not only for gene discovery but also to postulate possible mechanisms of expression regulation and control.

After LOWESS normalization, filtering by expression level and a Welch ANOVA analysis we found that the transcript abundance profile of 3,594 genes in the soybean cotyledons was significantly modulated when compared to the transcript profile observed within the imbibed seed at least in one of the time point measurements taken during seedling development (see Additional file 1: Complete collection of 3,594 genes with statistically significant data). In *Arabidopsis *the transcriptome of the imbibed seed is composed of mRNAs stored during seed development as well as the neosynthesized transcripts during water absortion [3]. In our study, the transcript profiles from stages 2–7 were compared to the already imbibed seed (48 hrs after initiation of imbibition). Classification by gene ontologies and cluster analysis by *k-means *of over and under expressed genes provided a broader view of gene expression and modulation in the soybean cotyledons during this process and more specifically during the cotyledonary functional transition from nutrient storage to photosynthesis. The already identified and characterized genes reported in the literature, validate and support our data while the unidentified genes represent excellent candidates for discovering new elements of significant importance involved in soybean seedling development. Because organisms have adopted a strategy of expressing batteries of genes under common regulatory mechanisms, the individual transcripts in the clusters presented are most likely regulated and controlled in a coordinate manner. All the results obtained have been deposited in the NCBI Gene Expression Omnibus (GEO) database as accession number GSE6534.

### Oligo microarray data validation

Our data provided a collection of 3,594 genes with statistically significant data with a p-value cut off of 0.05 and a 0.05 false discovery rate (FDR). Detailed examination of the genes found and their expression profiles allowed us to identify several genes that had been already associated with the process of seedling development in soybeans and other plant species as well as many undiscovered soybean genes. RNA gel blot analysis of ten known genes and three unknowns (see Table 2) correlated with the general expression profile obtained by microarray analysis data. Although the general expression profile was comparable by the two techniques, the actual quantitative values were in some instances dissimilar. This can be explained by two main reasons: first, the signal obtained by using a full length EST clone as a probe for RNA gel blot analysis captures the expression levels of family members or genes with close homologies while the microarray data analysis only represents the expression values of a particular gene family member, enzymatic isoform or protein subunit, represented by the 70-mer oligo designed. Second, the oligo microarray analysis compiles data from 8 different point measurements (four per Biological replicate) (see Figure 1), while in the RNA blot gel analysis, we only have one point measure from one biological replicate. Therefore, it is possible to find small discrepancies in the quantitative values obtained by using the two different methods. An alternative way to more precisely validate quantitatively the oligo microarray data was to design specific primers to amplify exclusively the oligo sequence used in the microarray study, and set up a qRT-PCR experiment. Data obtained by this method correlated with the data obtained by oligo microarray analysis since we were able to amplify specifically the same 70-mer oligo sequence used in the array, providing an excellent validation tool. We can still observe some point measurements discrepancies due to the fact that only one biological replicate was used for the qRT-PCR experiment.

### Role of the glyoxylate cycle during the functional transition

The functional transition of the soybean cotyledon initiates when the dry seed (yellow) is imbibed in water followed by a series of dramatic morphological and physiological changes. After imbibition is complete the soybean cotyledons increase their size by 2 to 3 times fold. Then, protrusion of the hypocotyl occurs followed by emergence, transition to photosynthetic activity (greening) and senescence [5]. Cotyledons are exposed to environmental changes such as water content, light exposure, temperature and nutrient concentrations. At the cellular level, the primary storage reserve in soybeans is in the form of triacylglycerol (TAG) that needs to be mobilized and utilized to support plant growth and development. It has been demonstrated in *Arabidopsis *that significant amounts of carbohydrates derived from stored lipids are required for post-germinative seedling development [13,14]. Our data shows that in soybean cotyledons a specific gene for lipase [Clone ID: Gm-c1013-3043] initially catalyses the release of fatty acids (FA) from TAG stored within oil bodies (see Figure 5). The fatty acids produced are activated to acyl-CoA esters and enter the glyoxysome. This single membrane organelle is a specialized form of peroxisomes where the acyl-CoA molecules are broken down to acetyl-CoA via the β-oxidation spiral. The acetyl-CoA then serves as a substrate for the subsequent reactions of the glyoxylate cycle which is an adapted form of the respiratory tricarboxylic acid cycle. Succinate is produced, transformed to malate in the mitochondrion and exported to the cytoplasm. Then, it is converted to carbohydrate which is translocated and used in the growing parts of the plant [12-15,43,55].

Accumulation of mRNA levels of enzymes involved in the glyoxylate cycle during seed germination and post-germinative development has been previously reported in *Brassica napus *[4] and *Arabidopsis *[3,6]. During the first stages of soybean seedling development and throughout the cotyledonary functional transition we found that genes corresponding to the specific isoforms of the key enzymes of the glyoxylate cycle [MDH – Clone ID: Gm-r1088-2431; MS – Clone ID: Gmr-1070-7761, Gmr-1070-8044 and Gm-r1088-2873; CS – Clone ID: Gmr-1070-1609; AH – Clone ID: Gm-c1027-1520, Gm-r1088-7889 and Gm-r1088-8860; ICL – Clone ID: Gm-r10883791] show high levels of expression (see Figure 5). After greening, the expression profile of all of these genes show a significant reduction in their levels of expression. These observations support the idea that the glyoxylate cycle is particularly active during the four initial stages of soybean germination and inactive during the photorespiratory phases.

Typically during the initial phases of germination the cotyledons are yellow, the chloroplasts are not fully developed and leaf peroxisomes are less abundant. Once the lipids are metabolized the cotyledons undergo greening to a point where the glyoxysomal enzymes decline while the ones characteristic of leaf peroxisomes become predominant (see Figure 5). Our data strongly supports the concept of a conversion from glyoxysomes to leaf peroxisomes during the functional transition of the cotyledonary cell. After greening the cotyledons undergo senescence and several studies have demonstrated that leaf peroxisomal activity disappears and glyoxysomal activity resumes [11,56]. We postulate that initially there is a predominant population of glyoxysomes that carry out lipid mobilization and utilization to produce carbohydrates necessary for the survival of the plant. Our data indicates that peroxisomes are also present during these initial phases by examination of the soybean specific gene expression profiles of typical enzymes associated with peroxisome activity like glycolate oxidase [Clone ID: Gm-c1020-110], glutamate aminotransferase [Clone ID: Gm-c1065-9456] and catalase [Clone ID: Gm-c1027-5248 and Gm-r1088-8229] (see Figure 5 and 6). Based on this information is reasonable to believe that throughout the functional transition of the cotyledonary cell in soybeans there is a co-existence of glyoxysomes and peroxisomes with their respective battery of enzymes. Also during this period our data suggests that chloroplasts are assembled and photosynthetic elements become abundant.

After greening of the cotyledon the glyoxysomal activity is highly reduced and the photorespiratory phase of the cotyledonary cell is favored (see Figure 6). During this time, the phosphoglycolate produced in the chloroplast through the Calvin cycle is mobilized to the peroxisome in the form of glycolate where is oxidized to glyoxylate in a reaction that yields hydrogen peroxide. Glycine is finally synthesized and transferred to the mitochondrion, converted to serine and returned to the chloroplast in the form of glycerate after several metabolic reactions within the peroxisome. Because photorespiration is an inevitable accompaniment of photosynthesis in most plants leaf peroxisomes are large and abundant in photosynthetic tissues. The toxic hydrogen peroxide produced is broken down to water and oxygen by the action of catalase [Clone ID: Gm-c1027-5248, Gm-r1088-8229] an enzyme that is as a marker for peroxisome activity (see Figure 6).

Along with genes involved in the glyoxylate cycle and in support of our hypothesis we also found genes with similar expression profiles involved in chloroplast assembly and function. For instance, chloroplast inner membrane import protein Tic22, chloroplast outer envelope protein 34, chloroplastic outer envelope membrane protein (OEP75), coproporphyrinogen oxidase, plastidic ATP/ADP transporter, putative plastid glucose 6 phosphate/phosphate translocator and 18 more chloroplast-associated genes. Apparently this group of genes associated by common chloroplast functions contains a significant number of genes that share a similar expression profiles. This observation suggests that this group of genes may play a co-ordinated role with the genes previously described during the onset of germination and throughout the functional transition. It's important to note also that there is a significant number of chloroplast-related genes with completely different expression profiles, suggesting alternative control and regulation processes and therefore different functions.

More interestingly, we found more than 60 specific soybean genes (see Additional file 1: Complete collection of 3,594 genes with statistically significant data) with unknown function and no homology with any other plant species genes in the data base that have a similar expression profile to the genes previously described in the glyoxylate pathway. Furthermore the glyoxylate cycle has been also associated with chlorophyll breakdown, opening the possibility of further unknown elements associated with this pathway in plants [13,57]. We believe that these soybean unknown genes represent a valuable source to discover and characterize new genes that most likely play an important biological role during the processes of cell division, lipid mobilization through the glyoxylate cycle and chloroplast assembly during seedling development and the functional transition. These genes also have the potential to be key elements in the regulatory and control mechanisms associated with these metabolic processes.

### General view of gene expression in cotyledons throughout seedling development

Although the identity of all the participants in the complex program involved during soybean seedling development and the cotyledonary functional transition is still unknown, here we present an assemblage of individual statistically significant elements including signals, regulators, inducers and precursors that may be key participants during this developmental phase in soybeans. For instance, auxins must play a critical role in cotyledon gene expression, since they are thought to regulate and influence diverse whole-plant responses such as tropisms, apical dominance, root initiation, and at the cellular level, mechanisms such as cell extension, division and differentiation. Auxins have been proven to up or down regulate numerous genes [58]. The auxin down regulated genes ADR6, ADR11 and ADR12 not only depend on auxin concentrations but also on plant developmental stage and on the organ/tissue involved [50]. While ADR6, ADR11 and ADS11-2 are over expressed during the entire time study (set 12 of cluster analysis), ADR12-2 starts with very low levels of expression during stages 2 and 3 to then become up-regulated during stages 4–7 (set 10 of cluster analysis). Although the function of these auxin down regulated genes is still unknown it is interesting to find that different auxin responsive elements function in an independent manner from each other in the cotyledon during this developmental process.

We also identified particular protein subunits that could play an important function during soybean seedling development. For instance, the soybean β-conglycinin protein contains 3 different subunits known as: α, α' and β. The larger subunits α and α' are rapidly degraded after imbibition of the seed producing new active polypeptides and after 6 days of growth the β subunit is also hydrolyzed [37]. Wilson *et al.*[37] proposed that the newly synthesized polypeptides might be involved in the photosynthetic function of the soybean cotyledon however the authors could not defined which subunits were involved. Our results show that the transcripts for the three subunits are down regulated during the first 4 stages of soybean germination which is consistent with the decreasing amounts of β-conglycinin found after imbibition. However, the α' subunit shows over-expression during the final two stages (see Table 2) defined here as the green photosynthetic stages (stages 6 and 7) (see Figure 1). Based on these observations we can speculate that the hydrolysis product of the α' subunit could be the actual β-conglycinin subunit that Wilson *et al.*[37] proposed to be involved during photosynthetic activity of the soybean cotyledon due to its high activity during the photosynthetic stages.

Several expression profiles of particular transcription factors were also found in all different clusters, making them good candidates to follow up and investigate their possible role in regulating gene transcription of some of the relevant elements associated with soybean cotyledons during seedling development and the functional transition. Similarly regulators, precursors, inducers and hormone related genes can be found in these sets and therefore could be associated with seedling development-relevant functions in the cotyledon. A total of 885 genes expressed in the soybean cotyledon and divided into the 12 sets obtained by cluster analysis are genes that have no homology to any other genes in the data base. We want to emphasize the importance of these genes as powerful starting points for researchers to study, investigate and discover novel unique and soybean specific cloned genes that can play a fundamental role during the biological mechanisms and processes occurring in the soybean cotyledon during seedling development and the functional transition.

## Conclusion

Our data demonstrates that the global transcript profile of the soybean cotyledon during seedling development is extremely active, highly regulated and dynamic. We were also able to discriminate the expression profiles of individual gene family members, enzymatic isoforms and protein subunits and group them accordingly to their involvement in different functional activities relevant to seedling development and the cotyledonary functional transition in soybean. This means that we identified the individual specific soybean genes that play an important role during seedling development and discriminate them from the similar ones that are inactive and therefore irrelevant for this physiological process. This information is a powerful tool for future studies to better understand regulation and control mechanisms involved in soybean seedling development. Initially, we observed down regulation of genes that were important for carrying the cellular functions required for previous developmental processes and were not required for germination, for instance, seed maturation and dehydration. Simultaneously, we can see high gene expression activity for genes involved in the processes of cell division, *de novo *synthesis of DNA and proteins, as well as chloroplast assembly and function. The transcript profiles also delineate the cellular mobilization of stored lipids via the glyoxylate cycle and the transition to primarily peroxisomal function. The clear correlation between the expression profiles of elements of the glyoxylate pathway with the developmental stages defined for the functional transition indicate that these elements play an essential role during this physiological process. Our data suggests that in the soybean cotyledon a very complex and synchronized system of control and regulation of several metabolic pathways is essential to carry out the necessary functions during seedling development and provides a novel set of cloned cDNA sequences with unknown function and high correlation with the cotyledonary functional transition.

## Methods

### Experimental design

Four *Glycine max *cultivar Williams dry seeds were planted per small pot (4 inches) containing pre-wetted Universal Mix SB300 soil in the green house. A total of 50 pots (200 seeds) were initially used to collect and pool 10 individual cotyledon samples per developmental stage defined. Plants were grown for approximately two weeks with regular watering. A biological replicate was set up with another 50 pots planted four months later to collect tissue in a similar way from each developmental stage. A total of seven developmental stages were defined, starting with the imbibed seed normally collected 48 hrs after planting. These stages were defined based on radicle and hypocotyl size, emergence time, coloration of the cotyledonary tissue, and size of the emerged seedling (see Figure 1). The imbibed cotyledon tissue was used as the reference time point to compare global gene expression from each one of the 6 more subsequent cotyledonary developmental stages defined. Two replicates of 70-mer oligo chips containing 19,200 features were hybridized to compare differential transcript abundance between each stage (2 through 7) and the reference (stage 1). The dyes were swapped in two experimental replicates to avoid potential dye bias for a total of 4 Oligo chips per biological replicate. Since two biological replicates were set up, a total of 8 chips were used to compare transcript abundance per germination stage defined (see Figure 1).

### RNA extraction and quantification

Cotyledons from 10 individual samples per developmental stage, were collected and pooled, washed several times with distilled water, frozen in liquid nitrogen and freeze-dried for 48 hours. These tissues were then ground to a fine powder with a mortar and pestle, mixed with 3 times the weight of autoclaved Sand, White Quartz -50 +70 Mesh (Sigma Chemical Co. St. Louis, MO) and further ground for one more minute. Total RNA was extracted from 400 mgs of cotyledonary tissue plus sand mixture by following a standard RNA extraction protocol using phenol purification and lithium chloride precipitation [59]. Four different RNA preparations from the same tissue mixture were pooled, visualized in a denaturing formaldehyde 1.2% agarose gel and quantified in a NanoDrop ND-1000 Spectrophotometer (NanoDrop Technologies, Wilmington, DE).

### Microarray hybridization, scanning and quantification

A set of 19,200 70-mer oligo sequences were synthesized by Illumina/Invitrogen (San Diego, CA) based on publicly available soybeans EST and mRNA sequences. They were printed on Corning (Acton, MA) GAPS II Slides using a Genetix QArray^2 ^Robot (Hampshire, UK). The 70-mer sequences and all the available information including GenBank accession numbers and clone ID's represented by 19,200 set of oligos printed has been deposited in the NCBI Gene Expression Omnibus (GEO) as platform GPL4635. A total of 20 μg of the reference stage 1 (imbibed seed) (see Figure 1) RNA was used to be labeled by reverse transcription with Cy3-dUTP and compared to 20 μg of RNA from each of stages 2–7 labeled by reverse transcription with Cy5-dUTP following the protocol by Hedge et al., [60]. The soybean oligo array slides were pre-hybridized, hybridized and washed as described by Vodkin et al., [20], with slight modifications. Basically the volume of the cDNA probe plus poly A mixture was increased to 40.0 μL, mixed with an equal amount of pre-warmed (42°C) 2× hybridization buffer (50% formamide, 10× SSC, 0.2% SDS) and finally pipetted between the pre-hybridized slide and the 22 × 60 mm cover slip (LifterSlip, Erie Scientific Company, Portsmouth, NH). The hybridized slides were scanned with a ScanArray Express Microarray Scanner, Packard Bioscience/PerkinElmer (Shelton, CT) and the fluorescence was quantified by using the ScanArray Gx/ProScanArray, Microarray Analysis System (Packard biosciences/Perkin Elmer, Shelton, CT) using a protocol defined by a corresponding GAL file previously generated. The quantitated GPR files were then transferred to GeneSpring 7.2 Agilent Technologies (Palo Alto, CA) for further analysis.

### Data normalization, filtering, statistical analysis and clustering

For microarray data analysis using GeneSpring 7.2, Agilent Technologies (Palo Alto, CA) we uploaded initially a soybean oligo genome array file containing all the relevant information for each one of the oligos represented in the array. A systematic name was defined and catalogued along with its corresponding EST accession number, clone ID, Blast X (cut off 10E-06), top hit GI number and its description, top hit *Arabidopsis *ID and its description, corresponding gene ontologies to top hits, and associated EST's numbers. This file was uploaded in GeneSpring 7.2 (Silicon Genetics, CA) and was used as the soybean oligo genome information for further analysis. Data from a total of 48 slides, containing the intensity values for the control and the signal channels as well as the flag values for present, absent or marginal spots was uploaded and sample attributes were defined. The data was initially transformed for dye swap for the appropriate slide samples and all the intensity values less than 0.01 or negative were converted to 0.01 to reset biologically irrelevant or otherwise unacceptable expression values. Time was defined as the only parameter to be taken into account for analysis and 6 different conditions were defined: one for each time point comparison defined. After background subtraction and eliminating all the spots flagged as marginal or absent, data was normalized using an intensity-dependent LOWESS (locally weighted scatter plot smoothing) normalization method [61] to minimize systematic non-biological differences and standardize chips for cross-array comparisons. Normalized data was then filtered on expression level to obtain a set of genes with expression values in at least one of the comparisons made. This set was then used for statistical analysis. A Welch ANOVA (parametric test, variances not assumed equal) was carried out with a p-value cut off 0.05, a false discovery rate (FDR) of 0.05 and between 0.1 and 100 fold expression value change. This group of genes were further filtered on expression level creating restrictions to define relevant genes at any particular time point as well as to find expression profiles of biological relevance for the study. All the results obtained by microarray analysis have been deposited in the NCBI Gene Expression Omnibus (GEO) database as accession number GSE6534.

Also all members of this collection which contain Gene Ontologies (GO) classification for plants information were classified accordingly while the ones without it were manually classified using the GO guidelines [25,26] (see Additional file 1: Complete collection of 3,594 genes with statistically significant data). Clustering analysis by *k-means *grouped genes with similar transcript abundance profiles during germination and emergence in a pre-defined number of clusters using standard correlation as similarity measure (see Figure 4).

### RNA blot hybridization analysis and quantitative RT-PCR

A total of 14 genes with relevant expression profiles from the Oligo array data were chosen to be validated by RNA gel blot analysis. Total RNA (10 μg) from one biological replicate of the reference tissue and all different stages was prepared in 6.5% formaldehyde and 50% formamide, electrophoresed on a 1.2% agarose, 1.1% formaldehyde gel and blotted onto a nitrocellulose membrane [62]. The blots were hybridized to radioactive probes [62] obtained from the soybean public EST collection that contained the specific 70-mer oligo sequence to be validated. These clones were hand picked form the EST collection available, grown on YT media, purified, confirmed by sequence analysis and radioactively labeled.

Similarly, purified total RNA (5 μg) from each developmental stage from one biological replicate was converted into first strand cDNA by reverse transcription. RNA was denatured at 65°C for 5 min in a 10 μL reaction containing 1 mM dNTPs, 0.5 μg/μL Oligo(dT)_12–18 _followed by chilling on ice for 1 min. After the addition of 9 μL of a mixture containing 2 μL of 10× RT buffer (Invitrogen, Carlsbad, CA), 4 μL of 25 mM MgCl_2 _and 2 μL of 0.1 M DTT the reaction was incubated at 42°C for two min and 1 μL of SuperScript RTII (Invitrogen, Carlsbad, CA) was added and incubated at 42°C for 50 min. The reaction was terminated by incubation at 70°C for 15 min and the remaining RNA was degraded by addition of 1 μL of RNAseH and incubated at 37°C for 20 min. cDNA was then quantified by PCR using primers designed to amplify a 80 bp fragment containing the specific oligo to be evaluated. PCR reactions were carried out in a total volume of 25 μL, containing 200 nM of each primer (forward and reverse), 2.0 μL of previously synthesized cDNA and 12.5 μL of FailSafe Green Real-Time PCR 2× Pre mix G (Epicentre Biotechnologies, Madison, WI). Reactions were amplified in a MJ Opticon 2.0 (BIO-RAD, Hercules CA) using 0.2 mL low profile white tubes with optically clear caps (BIO-RAD, Hercules CA) as follows: 94°C for 2 min followed by 40 cycles of 94°C for 30 sec, 55°C for 30 sec, 72°C for 30 sec. All reactions were conducted in triplicate. Quantification was based on a Ct value and relative expression of each stage compared to the reference imbibed seed tissue was calculated using the 2^-ΔΔCt ^method [63].

## Authors' contributions

DOG prepared and printed the oligo array chips, carried out experiments, performed statistical analysis and drafted the manuscript. LOV led development of the 19,200 70-mer soybean oligo set, coordinated the project and edited the manuscript.

## Supplementary Material

Additional file 1 Complete collection of 3,594 genes with statistically significant data. The data provided enumerates clone identifiers, p-value, gene ontology and cluster analysis classification, BlastX hit ID and description and individual ratios per time measurement for each one of the genes with statistical significant data found in this study. Click here for file
